# Synthesis and Characterization of Poly(Butylene Sebacate-*C*o-Terephthalate) Copolyesters with Pentaerythritol as Branching Agent

**DOI:** 10.3390/ijms25010055

**Published:** 2023-12-19

**Authors:** Hyunho Jang, Sangwoo Kwon, Sun Jong Kim, Young-Teck Kim, Su-il Park

**Affiliations:** 1 Department of Packaging, Yonsei University, Wonju 26493, Republic of Korea; whyhyun@naver.com (H.J.); ksw0089@naver.com (S.K.); 2 CJ Cheil Jedang WhiteBio—CJ Research Center, Woburn, MA 01801, USA; ssun0526@gmail.com; 3 Department of Sustainable Biomaterials, College of Natural Resources and Environment, Virginia Tech, Blacksburg, VA 24061, USA; ytkim@vt.edu

**Keywords:** PBSeT, biodegradable, aliphatic–aromatic random copolyester, branching, melt flow, enzymatic degradation test

## Abstract

Poly(butylene sebacate-co-terephthalate) (PBSeT) copolyesters are prepared by melt polymerization via two-step transesterification and polycondensation using pentaerythritol (PE) as a branching agent. The effects of the incorporated PE on its chemical, thermal, mechanical, and degradation properties, along with the rheological properties of its melt, are investigated. The highest molecular weight and intrinsic viscosity along with the lowest melt flow index were achieved at a PE content of 0.2 mol%, with minimal reduction in the tensile strength and the highest tear strength. The addition of PE did not significantly influence the thermal behavior and stability of the PBSeT copolyesters; however, the elongation at break decreased with increasing PE content. The sample with 0.2 mol% PE exhibited a higher storage modulus and loss modulus as well as a lower loss angle tangent than the other samples, indicating improved melt elasticity. The incorporation of more than 0.2 mol% PE enhanced the enzymatic degradation of copolyesters. In summary, including within 0.2 mol%, PE effectively improved both the processability-related characteristics and degradation properties of PBSeT copolyesters, suggesting their potential suitability for use in agricultural and packaging materials.

## 1. Introduction

As environmental pollution intensifies owing to the abuse of petroleum-based non-degradable plastics, biodegradable plastics have attracted attention as key alternatives. In particular, the representative commercialized biodegradable aliphatic polyesters poly(lactic acid) (PLA), poly(butylene succinate) (PBS), and poly(hydroxy alkenoates) are widely used as alternatives for numerous non-degradable plastics [1,2,3]. However, the application of aliphatic polyesters as flexible packaging materials is limited because of their poor physical and mechanical properties [4,5]. Highly ductile biodegradable copolyesters composed of aliphatic and aromatic units, including poly(butylene adipate-co-terephthalate) (PBAT) and poly(butylene succinate-co-terephthalate) (PBST), have therefore been developed for use as flexible films, such as single-use bags and mulching films [6,7,8]. Despite their advantageous mechanical properties, PBAT and PBST are fossil-based biodegradable polyesters and therefore fail to satisfy the growing demand for carbon footprint reduction. Recently, several studies on poly(butylene sebacate-co-terephthalate) (PBSeT), which is a bio-sebacic acid counterpart of PBAT, have been conducted [9,10]. PBSeT is a novel biodegradable aliphatic–aromatic copolyester that can address the demand for increasing biobased carbon content and has mechanical properties comparable to those of PBAT [11]. Therefore, PBSeT is a promising biodegradable alternative to PBAT in flexible packaging applications.

PBSeT exhibits an elongation at break of more than 850%, which is similar to that of low-density polyethylene; however, its low melting point and melt strength limit its film processability and thus prevent its widespread application [11,12]. In particular, polymers used in film processing require a high melt strength because they must be able to withstand the expansion and contraction of the melt, which are required to stably produce a film of uniform thickness [13,14]. A common method for improving the melt strength of polymers involves the incorporation of functional monomers, such as branching agents, to produce branched copolymers [14,15,16,17]. The formation of a branched polymer structure using a branching agent can alter the chain topology of the chain and the integrity of the segmental arrangement, which alters the rheological properties of the melt [13,18]. Moreover, long-chain branching may strengthen interchain entanglement in the melt or form a cross-linked network, thereby increasing the melt strength of the polymer [19,20,21]. Several studies have described the synthesis, mechanical and thermal properties, and rheology of biodegradable polyesters with branching agents, including PLA, PBS, PBST, PBAT, and PBSeT [10,14,22,23,24].

A functional monomer with multiple arms containing hydroxyl (-OH) and carboxyl (-COOH) end groups is a suitable branching agent for producing biodegradable copolyesters [17]. Functional tri- and tetra-arm monomers with such end groups are typical branching agents. Tri-arm branching agents include glycerol, trimethylolethane, trimethylolpropane, tris(hydroxymethyl)aminomethane, triethanolamine, and dimethylol propionic acid, while tetra-arm branching agents include pentaerythritol (PE), ethylenediaminetetraacetic acid (EDTA), and 1,2,3,4-butanetetracarboxylic acid (BTCA). Li et al. recently synthesized PBSeT copolyesters with tri-arm modifiers and reported that PBSeT copolyesters containing glycerol had a relatively high crystallinity, puncture resistance, melt strength, and elasticity, whereas their thermal behavior and stability were not significantly affected by the addition of the modifiers [25]. In addition, the effects of tetra-arm branching agents on the properties of biodegradable copolyesters have been studied [17,26]. PE, which has a symmetric structure with four hydroxyl (-OH) end groups, has higher primary hydroxyl reactivity and is also less expensive than other tetra-arm branching agents [14,27]. PBST copolyesters incorporated with PE exhibited superior melt strength and tensile moduli as well as higher crystallinity than PBST and PBST copolyesters incorporated with diglycidyl 1,2,3,6-tetrahydrophthalate [14,28]. In addition, all-atom simulations of poly(butylene adipate-co-butylene itaconate) (PBABI) copolyesters show that PBABI samples incorporated with PE exhibit a higher degree of crosslinking per unit volume than PBABI samples incorporated with EDTA or BTCA [17,26]. These results suggest that PE can be a promising branching agent for improving the film processability of biodegradable copolyesters by inducing a higher degree of branching to enhance their melt strength. However, no reports have been published on the effect of branching on the properties of PBSeT copolyesters with PE as a branching agent.

In this study, a series of PBSeT copolyesters with a sebacic acid-to-dimethyl terephthalate ratio of 6:4 was synthesized using different amounts of PE as the branching agent for achieving a high branching efficiency to improve the rheological properties of the PBSeT melt.

The objective of this study was to investigate the effect of PE on the thermal, mechanical, and rheological properties of PBSeT copolyesters to develop a PBSeT copolyester with a suitable melt strength combined with good mechanical properties for film processing.

## 2. Results

### 2.1. Synthesis and Structural Characterization of PBSeT

A series of PBSeT copolyesters were synthesized via a two-step transesterification and polycondensation process using PE as the branching agent. Titanium tetrabutoxide (TBT) was employed as a catalyst, and the loading content of PE was varied from 0.1 to 0.3 mol% with respect to the diacid. The synthetic conditions and results are summarized in Table 1. PE did not affect the transesterification time; however, the polycondensation time decreased with increasing PE content. The formation of branched structures during melt polymerization has been shown to reduce the polycondensation time [14,28]. Therefore, it was inferred that the addition of PE led to the formation of branched structures in PBSeT.

**Figure 1:**
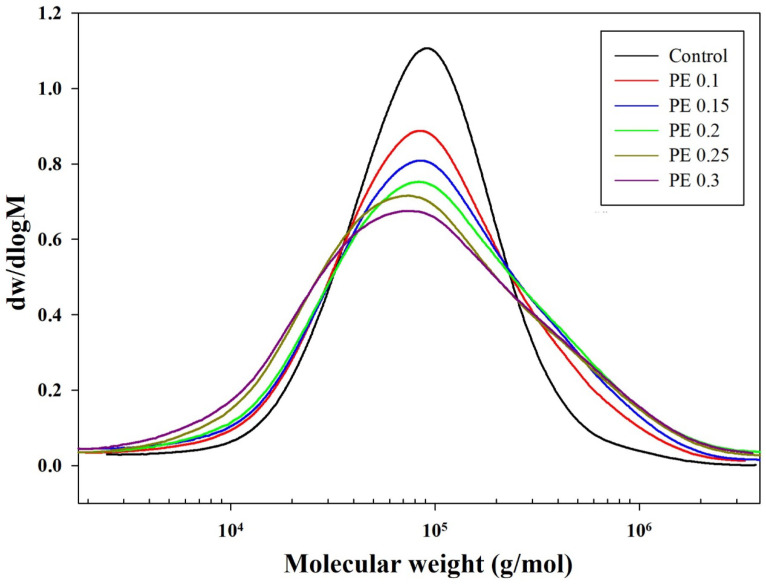
GPC curves of PBSeT copolyesters prepared with and without PE.

The GPC curves of the synthesized copolyesters are shown in Figure 1, and the molecular weight distribution parameters and polydispersity index (PDI) are summarized in Table 1. All PBSeT copolyesters had a unimodal molecular weight distribution (MWD) with an average molecular weight (M_w_) of >140,000 g/mol. Branched polymers exhibit higher molecular weights and PDI owing to the branching of polymer chains and increase in the branch length [28,29]. PBSeT copolyesters incorporated with PE had a broader MWD and higher PDI and M_w_ than the control as the linear PBSeT. The PDI of the PE-incorporated PBSeT copolyester increased gradually with increasing PE content, and the maximum M_w_ was achieved at 0.2 mol% PE. Notably, the high-molecular-weight content increased monotonically up to a PE content of 0.2 mol%, and as the PE content was increased further, the high-molecular-weight content decreased partially, whereas the low-molecular-weight content increased significantly. The increase in the content of the low-molecular-weight chains and high PDI resulted from an increased number of branching points and the consequent increase in short-chain branches owing to an excess of PE. Short-chain branches do not affect the melt flow, whereas long-chain branches that are sufficiently long to entangle with each other in the molten state significantly alter the flow properties of the melt [30,31]. The melting flow index (MFI) values of the PBSeT copolyesters with 0.25 and 0.3 mol% PE were higher than that of the sample with 0.2 mol% PE because of the relatively lower population of long-chain branches with high molecular weights. The changes in the intrinsic viscosity and relative average molecular weight of the PBSeT copolyesters were also similar. This is consistent with earlier observations that intrinsic viscosity is related to the molecular weight of the polymer and is not affected by the degree of branching or chain length [14,32].

**Figure 2:**
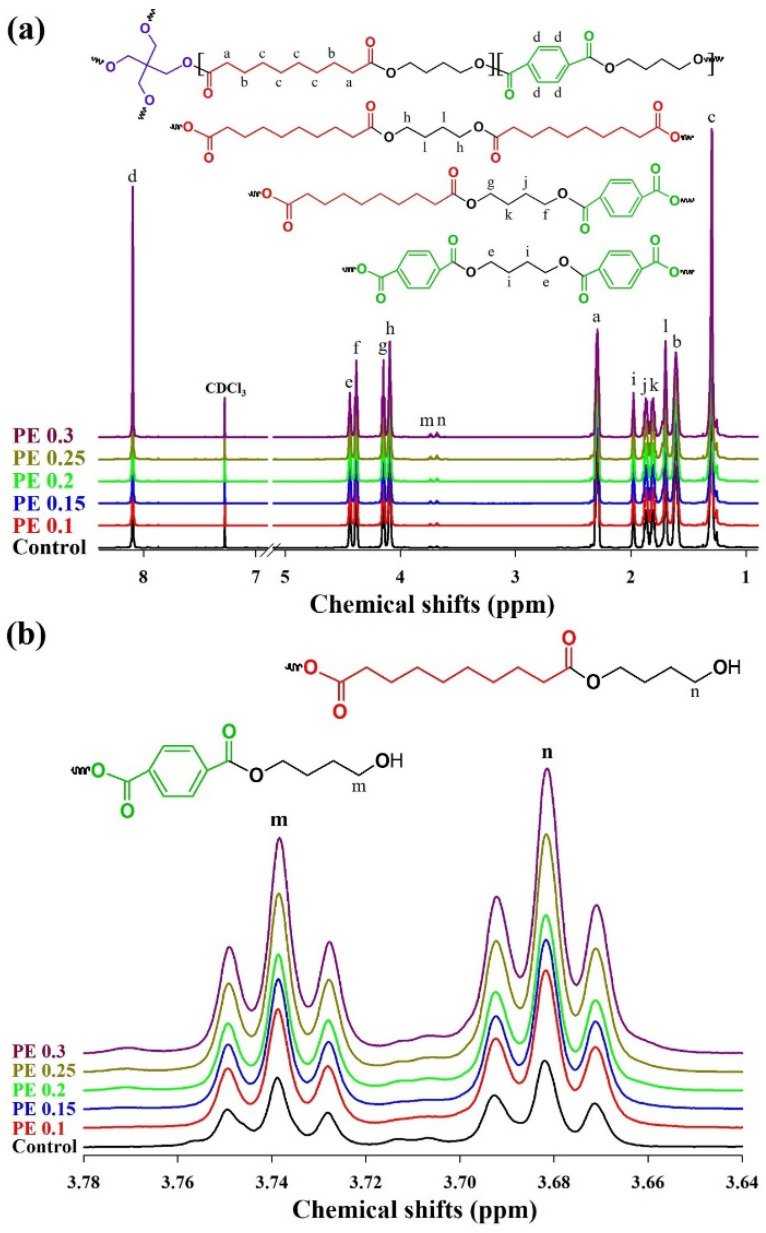
^1^H NMR spectra of PBSeT copolyesters prepared using PE as the branching agent: (**a**) full spectrum; (**b**) magnification of the spectrum from 3.64 to 3.78 ppm (-CH_2_OH region).

The chemical structures of the PBSeT copolyesters were analyzed by proton nuclear magnetic resonance (^1^H NMR) and Fourier transform infrared (FT-IR) spectroscopies. The ^1^H NMR spectrum of the PBSeT copolyesters (Figure 2) exhibit the characteristic signals of each unit and sequence type. The chemical shift in the deuterated chloroform solvent is located at 7.28 ppm. The characteristic signals corresponding to the same structural parts of the PBSeT copolyesters are consistent across all spectra. The chemical shifts in the benzene ring protons in the Se-B-T and T-B-T units are observed at 8.10 ppm. The signals corresponding to the protons of methylene (-CH_2_) groups in 1,4-butanediol (BDO; monomer) near and far from the oxygen atom are each split into four different signals at 1.98, 1.85, 1.83, and 1.70 ppm, and 4.44, 4.38, 4.15, and 4.09 ppm, respectively. These features of the chemical shift can be expressed by three possible triads with the BDO units: Se-B-SE, SE-B-T, and T-B-T [33]. Moreover, the chemical shifts in the poly(butylene sebacate) (PBSe) unit adjacent to the oxygen atom appear at 2.29 ppm, while those of another unit far away from the oxygen atom appear at 1.61 and 1.30 ppm. No signal corresponding to the methylene groups in PE could not be detected. Liu et al. reported that the chemical shift in the methylene groups in PE could not be identified because they overlapped with the signals of the methylene protons of BDO near the ester bonds; therefore, we were unable to quantify the degree of branching [14]. The two weak triplet signals at 3.74 and 3.68 ppm associated with methylene close to the terminal hydroxyl in the hydroxybutylene terephthalate and hydroxybutylene sebacate groups, respectively, are shown in Figure 2b. The signals corresponding to the terminal hydroxyl groups intensified with increasing PE content, evidently suggesting the occurrence of branching, although the degree of branching could not be quantified.

**Figure 3:**
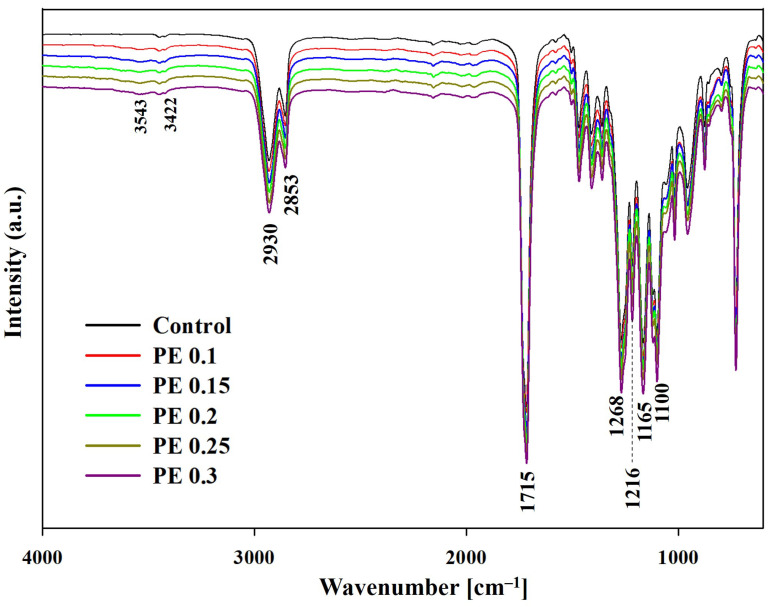
FT-IR spectra of PBSeT copolyesters with and without PE.

The FT-IR spectra of the PBSeT copolyesters synthesized with and without PE are presented in Figure 3. Absorption peaks typical of the aliphatic moieties in PBSe were detected at 2930 and 2853 cm^−1^; these correspond to asymmetric and symmetric C-H stretching vibrations, respectively [9]. Other absorption peaks characteristic of PBSeT, such as the stretching vibration of C=O at 1715 cm^−1^, stretching vibrations of C-O-C at 1100 and 1216 cm^−1^, stretching vibrations of aromatic C-O at 1268 and 1165 cm^−1^, and C-H stretching vibrations at 2930 and 2853 cm^−1^, were also observed [9,10,25,34,35]. Broad absorption peaks associated with the terminal hydroxyl groups were observed at 3628 and 3543 cm^−1^, while the peaks of the hydrogen-bonded hydroxyl groups appeared at 3448 and 3422 cm^−1^, as reported previously [17,25,34,35]. As the PE content in PBSeT increased, the intensities of the free and hydrogen-bonded hydroxyl peaks corresponding to hydroxyl end groups and hydrogen bonds tended to increase.

### 2.2. Thermal Transition of PBSeT Copolyesters

**Figure 4:**
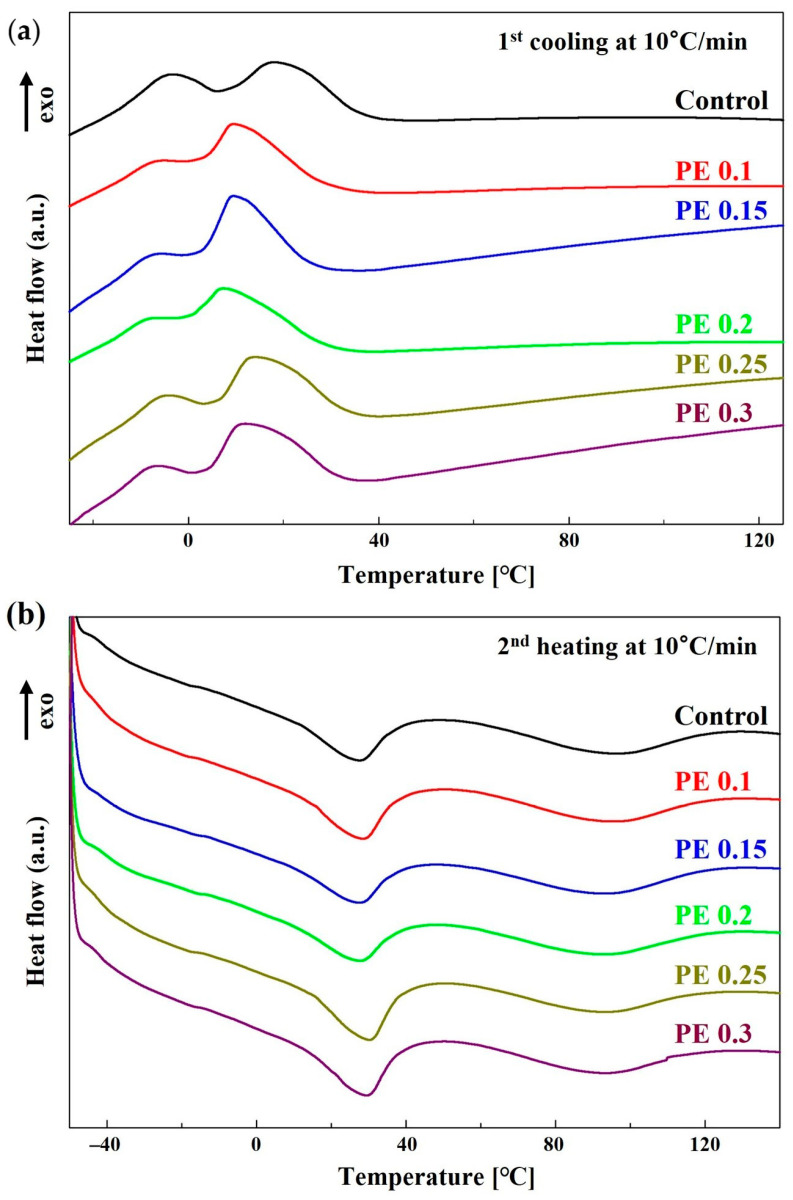
DSC thermograms of PBSeT copolyesters with PE as the branching agent: (**a**) first cooling scan and (**b**) second heating scan.

The thermal transition behaviors of the PBSeT copolyesters were investigated by differential scanning calorimetry (DSC) to determine the effect of branching induced by the incorporation of PE on the thermal characteristics of PBSeT. Figure 4 presents the DSC thermograms recorded during the first cooling and second heating cycles; the detailed thermal transition data are summarized in Table 2. The PBSeT copolyesters did not exhibit cold crystallization during the second heating cycle (heating rate: 10 °C/min), indicating the completion of melt crystallization. The first melting temperature (*T*_m1_) was dependent on the PE content, and showed a slight reduction from 29.4 to 26.6 °C. The second melting temperature (*T*_m2_) of the PE-incorporated PBSeT copolyesters increased slightly. The change in the degree of branching in the PBSeT based on the PE content did not have a significant effect on the *T*_m_ or melting peak position. All PBSeT copolyesters exhibited double crystallization peaks, regardless of whether or not PE was present during the cooling cycle. The crystallization temperature (*T*_c_) during cooling decreased with the introduction of PE, indicating that the branched structures in the PBSeT copolyesters delayed the crystallization of the copolymer. The degree of crystallinity (*X*_c_) first increased, and then, decreased with increasing PE content up to 0.2 mol%. Although branching can hinder segmental movement during crystal growth, the crystallinity of PBSeT was enhanced because the branching points acted as nucleating agents [14,36]. However, as the branch length increased, the crystallinity decreased because it becomes difficult to incorporate the chain in the crystalline structure [32,37]. The lowest crystallinity was observed at 0.2 mol% PE, which was attributed to the high population of relatively long-chain branches, as verified by GPC analysis.

**Figure 5:**
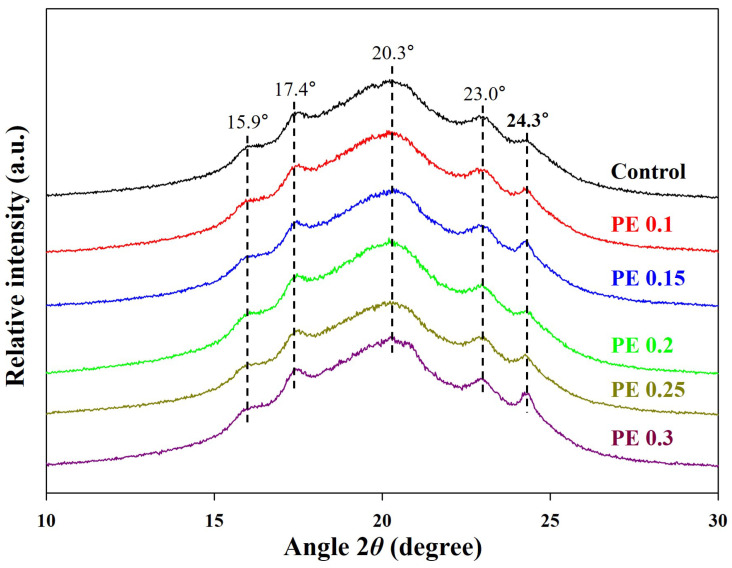
HR-XRD patterns of PBSeT copolyesters.

The crystal structure of the PBSeT copolyesters was identified via high-resolution X-ray diffraction (HR-XRD) (Figure 5). The main diffraction peaks in the spectra of the PBSeT copolyesters appear at 15.9°, 17.4°, 20.3°, 23.0°, and 24.3°, and are consistent with those reported in the literature [9,25,38]. Identical diffraction patterns were obtained for all samples, indicating that the crystal structure of PBSeT was not significantly affected by branching.

### 2.3. Thermal Stability of PBSeT Copolyesters

**Figure 6:**
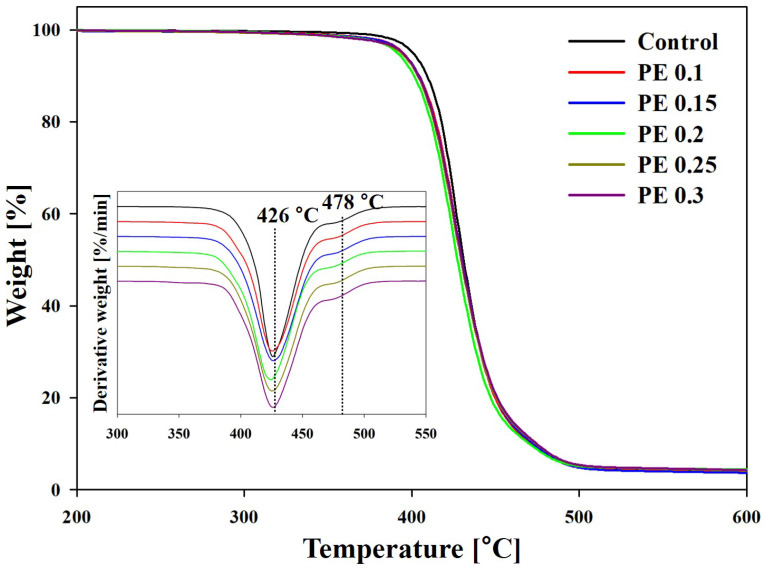
TGA and DTGA thermograms of PBSeT copolyesters.

The thermal stability of the PBSeT copolyesters was evaluated using a thermal gravimetric analyzer (TGA). The TGA and differential thermal gravimetric analysis (DTGA) thermograms are displayed in Figure 6, and Table 3 lists the thermal properties of the PBSet copolyesters. The thermal decomposition of the PBSeT copolyesters occurred in two steps, which is consistent with previous studies [9,25,34]. The onset decomposition temperatures (*T*_onset_) of the PE-incorporated PBSeT copolyesters were slightly lower than that of the control sample without PE owing to the lower molecular weights of branched PBSeT copolymers. However, the maximum thermal decomposition rate (*T*_max_), and the decomposition temperatures corresponding to weight losses of 50% and 75% (*T*_50_ and *T*_75_, respectively), were approximately similar, and all in excess of 400 °C, regardless of the PE content. The residual carbon content of PBSeT copolyesters at 600 °C was from 3.6 to 4.2%. We therefore concluded that branching does not affect the thermal stability of PBSeT, and the PBSeT copolyesters have sufficient thermal stability for film processing.

### 2.4. Mechanical Properties of Branched PBSeT Copolyesters

**Figure 7:**
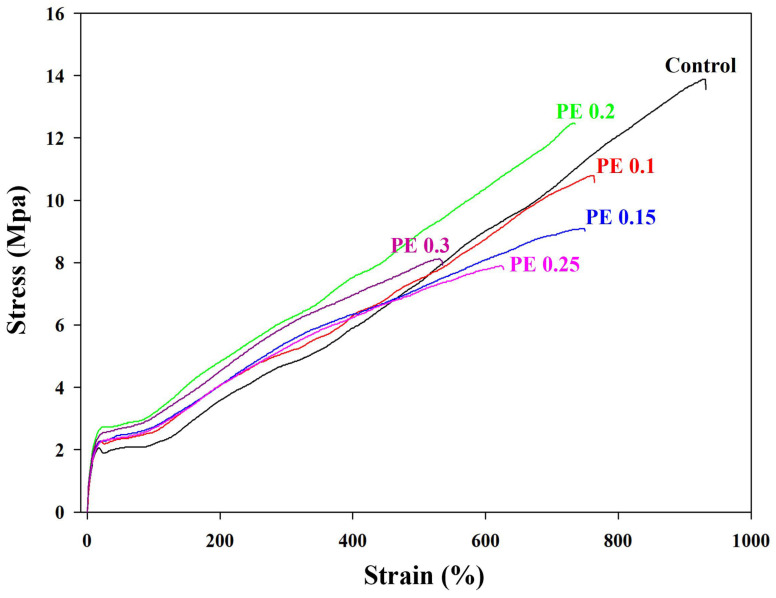
Stress–strain curves of PBSeT copolyesters with and without PE.

The dependence of the mechanical properties of PBSeT on the PE content was evaluated. The stress–strain curves are shown in Figure 7, and the detailed mechanical properties are presented in Table 4. The tensile strength of the PBSeT copolyester decreased upon the addition of PE, while the Young’s modulus increased. Numerous factors influence the tensile strength and Young’s modulus of a polymer, including the degree of branching and relative molecular weight and its distribution, composition, and crystallinity [25,39,40,41]. Owing to these complex factors, no apparent dependence of mechanical strength on the PE content was found; however, the elongation at break of the PE-incorporated PBSeT copolyester tended to decrease with increasing PE content. The increased probability of hydrogen bonding and chain entanglement due to branching hinders the rotation of the internal molecular chains and thereby reduces the flexibility of the polymer chains [14,28]. In other words, the increased number of branches at a higher PE content reduced the flexibility of the polymer chain, causing a reduction in the elongation at break of the PBSeT copolyester. On the contrary, the tear strength of the PBSeT copolyester with PE was improved compared to the PBSeT copolyester without PE. As the PE content increased, the tear strength of the copolyester first increased, and then, decreased, reaching its maximum at PE 0.2 mol%. Based on the effect of PE on the mechanical properties of PBSeT copolyesters, a PE content of 0.2 mol% was deemed suitable for film processing.

### 2.5. Rheological Properties of the Melts of PBSeT Copolyesters

**Figure 8:**
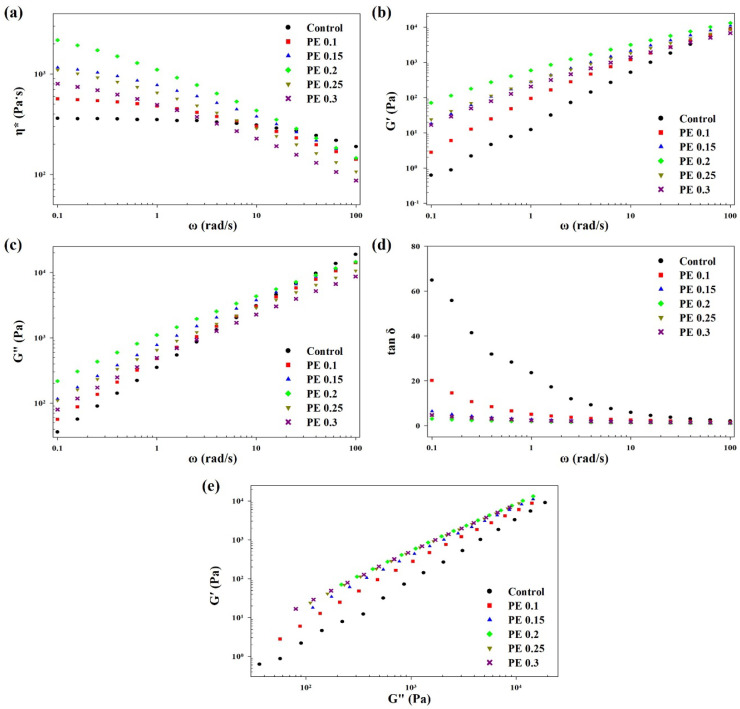
Rheological behaviors of PBSeT copolyesters at 120 °C: (**a**) complex viscosity (η*), (**b**) storage modulus (G′), (**c**) loss modulus (G″), (**d**) loss angle tangent (tan δ), and (**e**) Han plot (G′ vs. G″).

The rheological properties of polymer melts are key indicators of their melt strength and therefore determine the suitability of melts for film processing. The introduction of branched structures can change the topological structure of a polymer [42]. Long-chain branched polymers are known to have higher melt elasticity than linear polymers [14,28]. Therefore, the rheological characteristics of PBSeT copolyesters with and without PE, including their complex viscosity (η*), storage modulus (G′), loss modulus (G″), and loss angle tangent (tan δ), were investigated at 120 °C using a rotational rheometer. The complex viscosities are plotted as a function of angular frequency (ω) in Figure 8a. All PBSeT copolymers exhibited clear shear thinning behavior with increasing angular frequency. The PBSeT copolyester without PE (control) exhibited a Newtonian zone of complex viscosity. This feature is typical of linear polymers. The PE-containing PBSeT copolyesters did not exhibit a Newtonian zone in the measured angular frequency range. Such characteristics appear mainly in branched polymers, such as PLA and polypropylene, with long-chain branches [31,43]. Figure 8b,c show the storage and loss moduli of PBSeT copolyesters as a function of angular frequency from 10^−1^ to 10^2^ rad/s. G′ and G″ typically reflect the melt elasticity and melt viscosity, respectively, and are indicative of the viscoelastic behavior of the polymer melt. The G′ of the PBSeT copolyester reached a maximum value at 0.2 mol% PE, and then, decreased as the PE content further increased. However, both G′ and G″ decreased at lower angular frequencies, while the control and the copolymer with 0.2 mol% PE exhibited the highest and lowest slopes, respectively, of G′ and G″. The slope differed according to the PE content of the sample, but all PE-incorporated PBSeT copolyesters showed a lower slope than the control. The tan δ values (tan δ = G″/G′) are shown in Figure 8d; the tan δ in the low-frequency region first decreased as the PE content increased to 0.2 mol%, and increased thereafter. The observed tan δ (=G″/G′) implies a high storage modulus or a low loss modulus. These rheological behaviors suggest that the melt elasticity of PBSeT increased owing to branching induced by PE.

The rheological properties of a polymer could be affected by branching and also by its molecular weight and its distribution. The G′–G″ plot (known as the Han plot) is known to be related to the polydispersity of the polymer and the presence of long-chain branches in the polymer [28,44]. Figure 8e shows the Han plots of the PBSeT copolymers. At the same loss modulus, the storage modulus of the PBSeT copolyesters increased with increasing PE content over the entire angular frequency range. The PBSeT copolyesters containing more than 0.2 mol% PE exhibited similar Han plots, indicating that the Han plot was not affected by the molecular weight. In other words, PBSeT copolyesters with a PE concentration higher than 0.2 mol% have more long-chain branches and therefore have higher melt elasticity, as confirmed by the GPC data in the high-molecular-weight region. Consequently, a PE loading of ≤0.2 mol% is sufficient to improve the melt elasticity of the PBSeT copolyester for film processing.

### 2.6. Enzymatic Degradation Test of PBSeT Copolyesters

**Figure 9:**
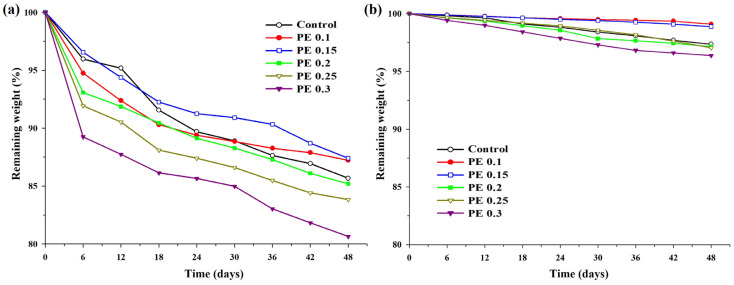
Enzymatic and hydrolytic degradation of PBSeT copolyesters: (**a**) remaining weight of PBSeT copolyesters under enzymatic degradation condition with *pseudomonas cepacia* lipase at 37 °C and (**b**) remaining weight of PBSeT copolyesters under neutral hydrolytic degradation condition (pH 7.4) at 37 °C.

**Figure 10:**
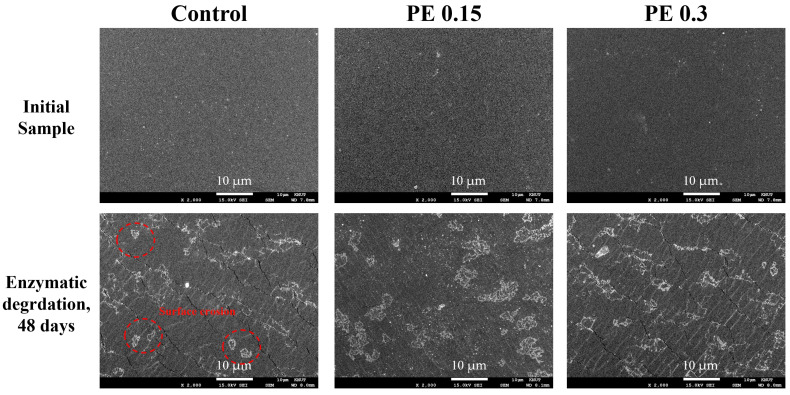
SEM images of PBSeT copolyesters before and after a 48-day enzymatic degradation test.

The study of enzymatic degradation behavior serves as a crucial indicator for indirectly assessing the biodegradability of bio-based biodegradable polyesters [45]. Enzymatic degradation tests were conducted to investigate the effect of introducing PE into PBSeT on the degradation behaviors under *pseudomonas cepacia* lipase in a phosphate buffer solution (pH = 7.4) at 37 °C. Figure 9 depicts the remaining sample weights over specific time intervals with and without the enzyme. Scanning electron micrographs of the surface of the PBSeT copolyesters before and after enzymatic degradation are displayed in Figure 10. Under enzymatic conditions, all samples exhibited obvious surface erosion, suggesting that degradation initiated at the polymer surface. After 48 days of incubation under neutral conditions (pH = 7.4) without enzymes, weight losses of approximately 3.6% were observed. Among these, PBSeT copolyesters with low PE content (<0.2 mol%) showed minimal weight loss, less than 1.2%. In contrast, under enzymatic conditions, there was substantial 12–19% weight loss, clearly indicating the effective involvement of lipase in the degradation process. When the PE content was less than 0.2 mol%, the weight loss of PBSeT copolyesters with and without the presence of enzymes gradually decreased due to their increased crystallites. This result is consistent with previous literature findings, suggesting that higher crystallinity restricts microorganism and water penetration, consequently reducing the enzymatic and hydrolytic degradation rates [10,46]. Conversely, PBSeT copolyesters with a high PE content (>0.2 mol%) exhibited enhanced degradation performance despite their high crystallinity. As discussed earlier, the excessive introduction of PE increases branching points in the polymer chain, leading to more short-chain branches and a population of low-molecular-weight chains. Consequently, the rapid degradation rate of the PBSeT copolyester with high PE content can be attributed to the degradation of branching points and low-molecular-weight short chains.

## 3. Materials and Methods

### 3.1. Materials

Sebacic acid (Se) and dimethyl terephthalate (DMT) were purchased from Daejung Chemical & Metals Co., Ltd. (Siheung, Republic of Korea). 1,4-Butanediol (BDO) was purchased from SK Chemicals (Seoul, Republic of Korea). Titanium tetrabutoxide (TBT), pentaerythritol (PE), phosphate buffer solution (pH = 7.4), and lipase from *pseudomonas cepacia* (>30 U/mg) were purchased from Sigma-Aldrich (Saint Louis, MO, USA).

### 3.2. Synthesis of PBSeT Copolyesters

**Figure 11:**
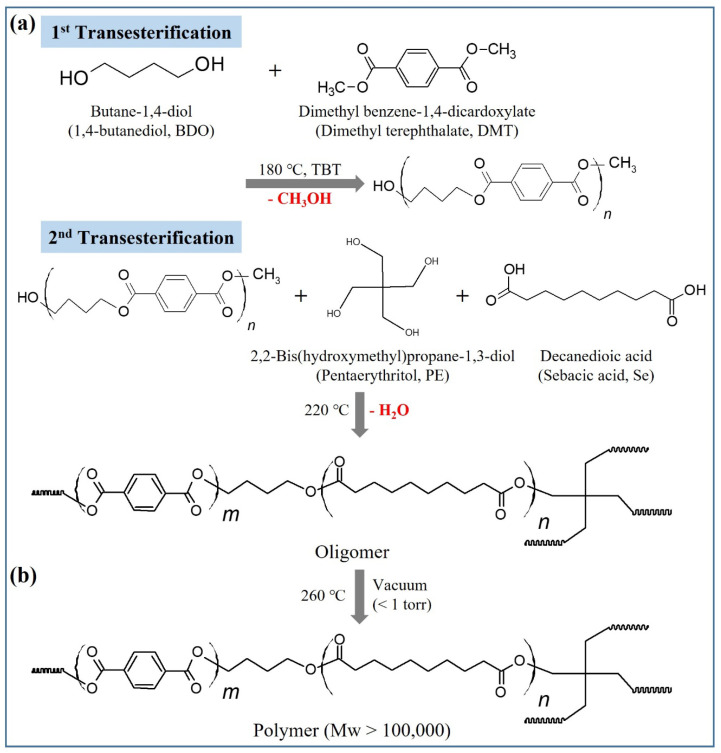
Procedure for the synthesis of PBSeT copolyesters using pentaerythritol as the branching agent: (**a**) step 1: transesterification, and (**b**) step 2: polycondensation.

Branched PBSeT copolyesters were synthesized using PE as a modifier over two steps (transesterification and polycondensation) in a 1 L three-necked reactor (Figure 11). Initially, BDO (225.3 g, 2.5 mol) and DMT (155.4 g, 0.4 mol) were transesterified with TBT (0.5 g, 1.5 mmol) as a catalyst at 180 °C under atmospheric pressure until the theoretical reaction methanol was discharged. After the first transesterification, Se (242.7 g, 1.2 mol) and PE were added, and the mixture was heated at 220 °C until the theoretical reaction water was discharged. Subsequently, polycondensation was carried out at 260 °C under vacuum. The molar ratio of [OH]/[COOH] was set at 1.25/1, and PE was introduced at concentrations of 0, 0.1, 0.15, 0.2, 0.25, and 0.3 mol% relative to the dicarboxylic acid.

### 3.3. Characterization of PBSeT Copolyesters

The weight-averaged molecular weight (M_w_), number-averaged molecular weight (M_n_), and polydispersity index (PDI, Đ) of each PBSeT sample was determined by gel permeation chromatography (GPC, Agilent Technologies 1200 series, Santa Clara, CA, USA) equipped with a refractive index detector. Two PLgel mixed-C columns and a PLgel 10 μm guard column were used. Each sample was dissolved in chloroform at a concentration of 2.0 mg/mL and filtered through a PTFE membrane (pore size: 0.45 μm). Poly(styrene) standards were used for calibration, and GPC was performed using a chloroform eluent at a flow rate of 1.0 mL/min at 30 °C with an injection volume of 200 μL. The intrinsic viscosity (IV) was estimated with an Ubbelohde viscometer in a water bath at 25 °C using a solution of PBSeT (0.125 g) in chloroform (25 mL) and calculated by following the Billmeyer equation [47]:

[η] = 0.25 (η_rel_ − 1 + 3 ln η_rel_)/*C* (1)

where [η] is the intrinsic viscosity, η_rel_ the relative viscosity, and *C* is the polymer solution concentration (g/dL).

The melt flow index (MFI) of each PBSeT copolyester was measured using a QM280 melt flow indexer at 170 °C with a load of 2.16 kg. The MFI of each sample was presented as the average of at least five samples with standard deviation. The ^1^H NMR spectra of the copolyesters were analyzed to identify the chemical structures with a BRUKER-AVANCE III 600 MHz spectrometer (Bruker, Karlsruhe, Germany). Deuterated chloroform and tetramethyl silane were used as the solvent and internal reference, respectively. Fourier transform infrared (FT-IR) spectra of the samples were recorded using a Spectrum 65 FT-IR spectrometer (PerkinElmer, Inc., Waltham, MA, USA) at room temperature in a wavenumber range from 4000 to 400 cm^−1^ over 32 scans and at a 2 cm^−1^ resolution.

Thermal transition behaviors were investigated by differential scanning calorimetry (DSC, DSC-Q20 calorimeter, TA Instruments, Milford, MA, USA). A sample (approximately 5–6 mg) was sealed in an aluminum pan heated and cooled at a rate of 10 °C/min from −50 to 150 °C under nitrogen flow. The melting temperature (T_m_), glass transition temperature (T_g_), and crystallization temperature (T_c_) were determined by the second heating scan and first cooling scan, respectively. The crystal structure was characterized by high-resolution X-ray diffraction (HR-XRD, SmartLab, Rigaku, Japan) with a Cu Kα source (1.5406 Å). All samples were scanned from 2θ = 10° to 2θ = 50° with a step size of 0.02° and a dwell time of 30 s per step.

A thermal gravimetric analyzer (PerkinElmer, Waltham, MA, USA) was used to determine the thermal stabilities of the samples. The PBSeT copolyester samples (10 mg) were heated from 30 to 700 °C at a heating rate of 10 °C/min under nitrogen flow.

The mechanical properties of the PBSeT copolyesters were analyzed using a universal testing machine (Qumesys, Anyang, Republic of Korea) at a tensile speed of 200 mm/min, in accordance with the ISO 527 standard. Dumb-bell shaped specimens were prepared via hot melt compression molding at 160 °C and 25 MPa for 2 min. At least five specimens of each sample (115 mm × 25 mm × 0.28 mm) were measured, and the values are presented as the mean and standard deviation.

The tear strengths of PBSeT copolyesters were tested according to ISO 6383-1:2015 using a universal testing machine (Qumesys, Anyang, Republic of Korea). The test specimen had a size 150 mm × 50 mm with a notch, whose depth was 75 mm and which was located in the center of specimen. At least five specimens of each sample were tested with the tearing rate at constant 200 ± 10 mm/min.

The rheological characteristics of the melts were evaluated using a TA-AR2000EX rotational rheometer (TA Instruments, Milford, MA, USA) using a cone plate type with a diameter of 25 mm and gap of 1.1 mm at 120 °C. The strain was set to 1% in the angular frequency range of 0.1–100 rad/s for the measurements of complex viscosity, storage modulus, and loss modulus.

### 3.4. Enzymatic Degradation Test of PBSeT Copolyesters

Hot-pressed films with dimensions of 10 × 10 × 0.2 mm^3^ were prepared for both hydrolytic and enzymatic degradation tests. The PBSeT copolyester films were placed in a 5 mL vial containing 3 mL of a phosphate buffer solution (pH = 7.4) and a phosphate buffer solution (pH = 7.4) with 0.5 mg/mL *pseudomonas cepacia* lipase. All degradation samples were incubated in a water bath at 37 °C for 48 days. The degradation solutions were replaced every week, and then, the PBSeT film samples were rinsed with distilled water and dried before weighing. The PBSeT copolyester film samples after the degradation test were weighed to determine the degree of degradation and characterized using a scanning electron microscope (SEM).

All data were collected from at least three samples, and the values are presented using descriptive statistical analysis based on the mean and standard deviation, calculated using Microsoft Excel for Office 365 MSO (version 2311).

## 4. Conclusions

Branched PBSeT copolyesters were synthesized through a two-step transesterification process followed by polycondensation using PE as a branching agent. The degree of branching and MWD of the PBSeT copolyesters increased with increasing PE, resulting in superior crystallinity than that of the linear PBSeT copolyester. The PE-branched PBSeT copolyesters exhibited enhanced tensile moduli, and had sufficient thermal stability for application in film processing; however, a change in the thermal stability of PBSeT owing to branching could not be confirmed. The rheological properties of the branched PBSeT copolyesters were greatly superior to those of their linear counterparts. In particular, the lowest MFI, highest melt elasticity, and largest degree of long-chain branching were achieved with a PE concentration of 0.2 mol%. The rheological properties of copolymers with higher PE contents (>0.2 mol%) deteriorated owing to the higher number of low-molecular-weight components that resulted from excessive branching. In contrast, the enzymatic degradation properties of PBSeT copolyester were enhanced, with PE content of more than 0.2 mol%. Overall, appropriate PE loading (within 0.2 mol%) is optimal for enhancing the melt strength and consequently improving the film processability, as well as enhancing the degradation property, of PBSeT copolyester, which is a promising biodegradable packaging material.

## Figures and Tables

**Table 1:** Synthetic conditions ^a^ and properties of PBSeT copolyesters with pentaerythritol (PE) as a branching agent.

Sample	ϕ_PE_ (%) ^b^	t_es_ (min) ^c^	t_pc_ (min) ^d^	^1^H NMR	[η] (dL/g) ^e^	GPC			MFI ^h^ (g/10 min)
				ϕ_BT_ (%)		M_n_ (g/mol) ^f^	M_w_ (g/mol) ^f^	Đ ^g^	
Control	0	110	360	42.7	1.19	50,500	143,000	2.84	33.3
PE 0.1	0.1	110	350	42.4	1.23	39,100	158,000	4.04	26.7
PE 0.15	0.15	110	325	42.5	1.30	37,500	178,000	4.74	18.7
PE 0.2	0.2	110	285	42.5	1.58	37,800	211,000	5.57	7.3
PE 0.25	0.25	110	255	42.3	1.42	32,000	194,000	6.06	20.1
PE 0.3	0.3	110	250	42.5	1.32	29,100	189,000	6.49	21.7

^a^ Transesterification (ES) temperatures: 1st ES (180 °C), 2nd (220 °C); polycondensation temperature: 260 °C. ^b^ Molar percentage of pentaerythritol based on the dicarboxylic acid. ^c^ Transesterification time. ^d^ Melt polycondensation time. ^e^ Intrinsic viscosity measured at 25 °C using chloroform as solvent, calculated based on Billmeyer equation. ^f^ M_n_, and M_w_ determined by GPC in chloroform at 30 °C against polystyrene standard. ^g^ Polydispersity index (PDI) calculated by M_w_/M_n_. ^h^ Melting flow index (MFI) measured at 170 °C with a load of 2.16 kg.

**Table 2:** Thermal transition properties of PBSeT copolyesters.

Sample	Thermal Transition (°C)					ΔH_m_ (J/g)			ΔH_c_ (J/g)			X_c_ (%) ^a^
	T_g_	T_m1_	T_m2_	T_c1_	T_c2_							
Control	−41	28	95	18	−3	6.2	/	9.1	10.3	/	5.5	13.6
PE 0.1	−42	29	94	10	−5	7.5	/	9.0	12.0	/	4.0	14.0
PE 0.15	−41	28	93	9	−6	5.8	/	9.3	13.0	/	4.0	15.6
PE 0.2	−40	28	93	8	−8	5.5	/	9.0	10.8	/	2.6	13.2
PE 0.25	−42	29	93	14	−4	8.1	/	7.9	12.1	/	4.3	15.2
PE 0.3	−42	29	93	12	−7	8.1	/	8.6	13.4	/	5.6	17.0

^a^ Crystallinities of PBSeT copolyesters were calculated by HR-XRD method using Origin Pro 2018 software; crystallinity (%) = [(area under the crystalline peaks)/(area under all peaks)] × 100.

**Table 3:** Thermal stabilities of PBSeT copolyesters.

Sample	*T*_onset_ (°C)	*T*_50_ ^a^ (°C)	*T*_75_ (°C)	*T*_max_ (°C)		Residue at 600 °C (%)
				Step 1	Step 2	
Control	411	431	445	426	478	4.0
PE 0.1	407	430	444	429	476	3.6
PE 0.15	406	430	445	426	478	3.6
PE 0.2	405	428	444	424	476	3.9
PE 0.25	405	429	445	425	476	4.0
PE 0.3	406	430	446	427	477	4.2

^a^ The temperature at weight loss (%).

**Table 4:** Mechanical properties of PBSeT copolyesters.

Sample	Young’s Modulus (MPa)	Tensile Strength (MPa)	Tear Strength (N/mm)	Elongation at Break (%)
Control	20.8 ± 0.6	14.7 ± 0.2	50.9 ± 1.0	917.6 ± 26.4
PE 0.1	22.0 ± 0.9	11.5 ± 0.1	57.1 ± 1.7	756.5 ± 10.2
PE 0.15	22.1 ± 0.9	8.9 ± 0.2	59.5 ± 1.5	739.2 ± 11.6
PE 0.2	25.6 ± 0.8	12.2 ± 0.2	61.9 ± 1.1	725.4 ± 19.5
PE 0.25	21.8 ± 0.9	8.0 ± 0.1	56.3 ± 1.6	638.9 ±11.6
PE 0.3	24.4 ± 1.0	8.4 ± 0.2	53.3 ± 1.4	566.9 ± 34.5

## Data Availability

Data are contained within the article.
